# The Fate of the Infirm

**Published:** 1896-10-10

**Authors:** 


### The Fate of the Infirm.

Much has been heard of late reg rding the improvements which have taken place in the treatment of the sick in workhouse infirmaries, and no doubt in many unions, especially those situated in the metropolis or in the larger towns, their lot is now a much happier one than it was some years ago, although still in a large number of places the treatment of the pauper sick is far from being what it ought to be. The improvement in the treatment of those who are really ill has, however, only served to emphasize the more the hardships which are endured by those who, while old and feeble, are as yet not suffering from actual disease. These people are left in the workhouses to bear the rude buffetings of fortune and of workhouse rules as best they may, until the time arrives when sickness, blessed sickness in cases, where there is a separate infirmary, carries them into that haven of rest, where they are nursed, and fed, and looked after as befits their weakness and infirmities.

In truth, as matters stand at present in many unions, the lot of the aged and infirm is a far more pitiable one than that of the sick, and it is strange that the sympathy which has been so freely displayed towards those who are cast down by disease should have skipped so lightly over those who are merely wearing out their lives as "aged and infirm." Perhaps the explanation is to be found in the fact that reform in workhouses has largely sprung from the medical and nursing side rather than from purely official quarters; in any case, however, it would seem that, useful as the reforms which have been carried out have been, they have tended somewhat to leave in the ditch, to leave even in worse plight than before, those classes which just missed the hand of the reformer. The aged and infirm form a class in which some halt but a short time in their progress towards the grave—some halt for long. They form a class whose natural affinities generally are with, and whose end always is in, the sick ward; and with the sick, in olden times, they were more or less allied. But now that the appliances for dealing with disease are mostly swept away into the grand, new, separate infirmaries, these ancient people, these feeble folk, are left stranded in the workhouses, where they have to bide their time in buildings planned, and under officials appointed, to be a terror to idle vagabonds.

The Poplar Union has a large separate infirmary, but like many others it has certain sick wards and lunatic wards still attached to its workhouse, where, as in all unions, the aged and infirm also are lodged. Here an old woman, Mary Pendred by name, was an inmate. According to the account given at the inquest which was held in regard to her death, it appears that, being taken ill, she was removed to the sick ward, a few days after which she became "violent"; in other words, "she dressed herself and said she was going out of the workhouse, and made a great noise, disturbing the other patients." By order of the medical officer she was removed to the lunatic ward—the padded room—where she died four days afterwards. Evidence was given to the effect that the patient had complained of having been ill-used and thrown violently on the floor. On this, however, we do not feel inclined to lay much stress. It is fairly certain that a peaceable old lady of eighty-five would hardly have been likely to have become unruly unless suffering from delirium caused by some bodily ailment, and what would cause delirious actions might well cause hallucinations and delusions. The point, however, on which we would lay stress is, that this patient ought to have been removed to a properly-appointed infirmary. If she had had the good luck to be taken ill gradually, so as to have been drafted off to the separate infirmary, we may be quite sure she would not have been placed in the "padded-room"; and herein we see that the removal of the sick to separate infirmaries (places on which are concentrated such manifestations of humanity as the guardians care to display towards their charges) has left the aged and infirm, for whose use infirmaries are no longer meant, in almost worse plight than before.

